# Policy parameters for optimising hospital ePrescribing: An exploratory literature review of selected countries of the Organisation for Economic Co-operation and Development

**DOI:** 10.1177/20552076221085074

**Published:** 2022-03-21

**Authors:** Uditha T Perera, Catherine Heeney, Aziz Sheikh

**Affiliations:** Usher Institute of Population Health Sciences and Informatics, 172239 University of Edinburgh, UK

**Keywords:** Optimisation of ePrescribing, ePrescribing policy, hospital medicines management, digital maturity, national strategies, OECD health policy

## Abstract

**Objective:**

Electronic prescribing systems offer considerable opportunities to enhance the safety, effectiveness and efficiency of prescribing and medicines management decisions but, despite considerable investments in health IT infrastructure and healthcare professional training, realising these benefits continues to prove challenging. How systems are customised and configured to achieve optimal functionality is an increasing focus for policymakers. We sought to develop an overview of the policy landscape currently supporting optimisation of hospital ePrescribing systems in economically developed countries with a view to deriving lessons for the United Kingdom (UK).

**Methods:**

We conducted a review of research literature and policy documents pertaining to optimisation of ePrescribing within hospitals across Organisation for Economic Co-operation and Development (OECD) countries on Embase, Medline, National Institute for Health (NIH), Google Scholar databases from 2010 to 2020 and the websites of organisations with international and national health policy interests in digital health and ePrescribing. We designed a typology of policies targeting optimisation of ePrescribing systems that provides an overview of evidence relating to the level at which policy is set, the aims and the barriers encountered in enacting these policies.

**Results:**

Our database searches retrieved 11 relevant articles and other web resources mainly from North America and Western Europe. We identified very few countries with a national level strategy for optimisation of ePrescribing in hospitals. There were hotspots of digital maturity in relation to ePrescribing at institutional, specialisation, regional and national levels in the US and Europe. We noted that such countries with digital maturity fostered innovations such as patient involvement.

**Conclusions:**

We found that, whilst helpful to achieve certain aims, coordinated strategies within and across countries for optimisation of ePrescribing systems are rare, even in countries with well-established ePrescribing and digital health infrastructures. There is at present little policy focus on maximising the utility of ePrescribing systems.

## Background

In recent decades, the majority of economically developed countries have begun to implement digital systems within hospitals with the aim of improving the appropriateness of prescribing and medicines management decisions, reducing costs and enhancing patient safety.^1–3^ ePrescribing, a term used extensively in the United Kingdom (UK) context, has been defined as ‘the utilisation of electronic systems to facilitate and enhance the communication of a prescription or medicine order, aiding the choice, administration and supply of a medicine through knowledge and decision support and providing a robust audit trail for the entire medicines use process’.^4^ This definition is broader than similar terms, such as Computerised Physician Order Entry (CPOE) in the United States (US), which has been described as a ‘… a variety of computer-based systems that share the common features of automating the medication ordering process and that ensure standardised, legible, and complete orders’.^5^

**Figure 1.**
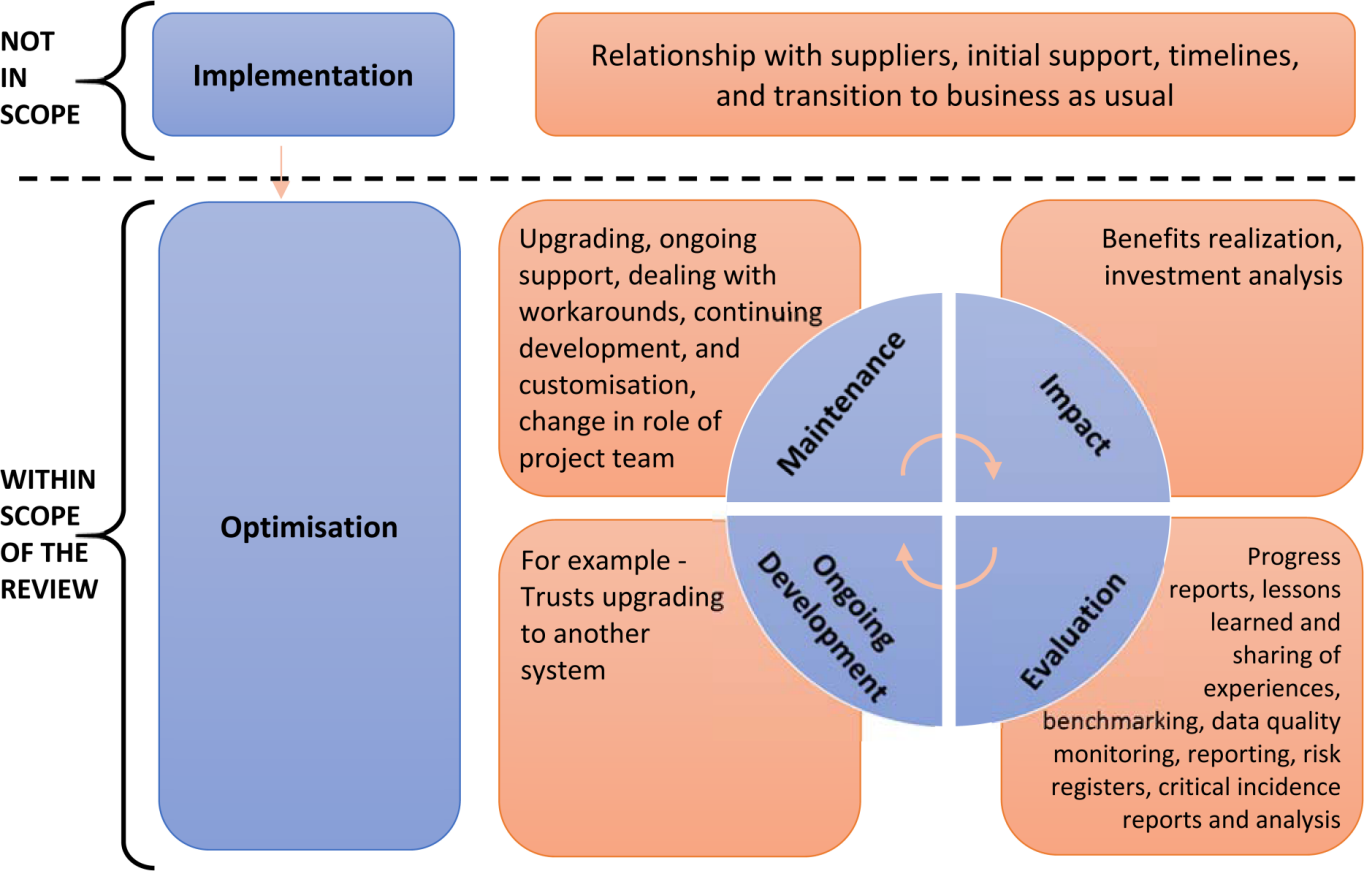
Scope of optimising ePrescribing project.^10^

ePrescribing systems have promised much in terms of safety, cost efficiency and integration of relevant data into the clinical decision-making processes, which has led to significant investment from governments and health systems.^6^ For example, the UK government has over the last decade injected substantial resources to help National Health Service (NHS) hospitals to procure and implement ePrescribing solutions.^7^ This is because there is now a growing body of work, which demonstrates that the introduction of an ePrescribing system does not in itself guarantee that promised benefits will materialise and that these systems may in some cases introduce new safety threats.^6,8,9^ For this reason, we concentrate here on policies that seek to improve existing ePrescribing systems (Figure 1).

It was noted by an independent report carried out for the Department of Health by Lord Carter of Coles in 2016 that hospital trusts vary enormously in relation to making efficient and effective use of ePrescribing technologies.^11^ Clear shared policy could potentially ensure any improvements made to local systems are taking place in an efficient, cost-effective and reproducible manner.^6^ Considering the continuing commitment to digital health in the UK, it is imperative that ePrescribing systems are not only implemented, but appropriately optimised to deliver the benefits envisaged by policymakers.

**Figure 3.**
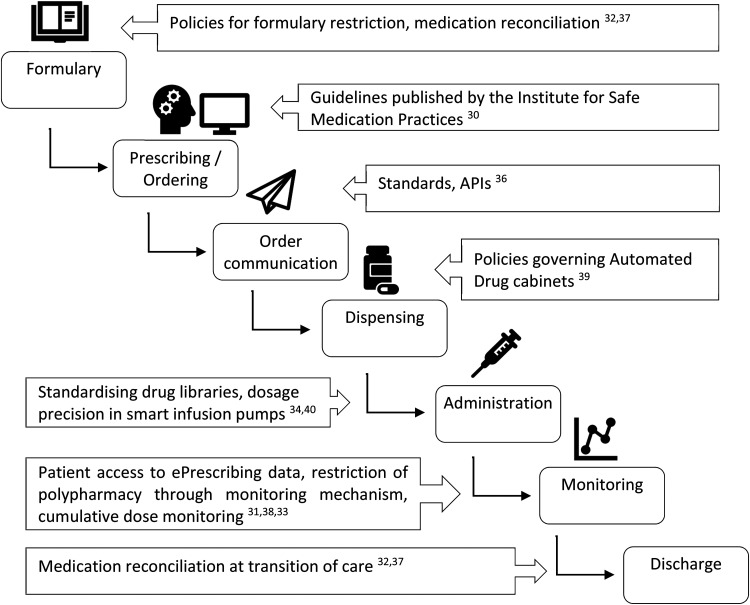
Focus of policy interventions in medication management process, adapted from Adeola et al.^39^

Optimisation activities can occur at any stage of the medicines management process, from defining the drug formulary through to dispensing and monitoring (Figure 3). These can also aim at producing better functionality from the IT system and improving user capabilities.^8^ Systems optimisation in the context of health IT has been described as ‘the organisational efforts to maximise the benefits and minimise the risks of utilising digital infrastructure to plan and deliver care’.^12^ Optimisation assumes previous implementation and some level of digital maturity. Different measures of digital maturity vary in what is captured.^13^ The National Health Service (NHS) currently measure digitally maturity broadly in terms of readiness to plan and deploy digital services, capabilities in using digital technology to support the delivery of care and infrastructure in place to support these capabilities.^14^

Noted drivers for the uptake of ePrescribing systems have included funding, regional and national policy for health IT, as well as patient safety guidance.^6,15,16^ The US Health Information Technology for Economic and Clinical Health (HITECH) Act 2009 was a national policy initiative for (amongst other things) the implementation of a digital strategy for medication management. In Europe, the Smart Open Services for European Patients (epSOS) project ran for 6 years from 2008. The aims of this programme included developing, piloting and evaluating cross-border sharing of information within European eHealth services, including ePrescriptions.^17^ These programmes have not ultimately led to consistent adoption of ePrescribing practices across the US or Europe. Nevertheless, the recognition of the potential benefits of having a coordinated approach to policy around ePrescribing continues. Attempts at standardisation can aid coordination across health systems and specialisations. For example, the internationally recognised Systematised Nomenclature of Medicine Clinical Terms (SNOMED-CT) has been crucial in advanced optimisation, such as the integration of genetic tests into Clinical Decision Support Systems (CDSS).^18^

Whilst ePrescribing is intended to allow effective and efficient use of health data to improve access to relevant information and to improve safety, the context into which these systems are being introduced is often characterised by fragmented political and payer infrastructures.^19^ Having a national level approach to the improvements within eHealth initiatives generally, potentially confers an advantage in terms of developing standards and a clear position in relation to the vendors of ePrescribing systems.^6^ There have also been calls for an international approach to health data governance, including ePrescribing.^20^ However, we did not find widespread evidence of such ‘macro’ level approaches.^15^ As we will discuss below, there are many examples of targeted policies for the optimisation of hospital ePrescribing systems at meso- or micro-levels, such as hospital site or specialisation specific. These sites apparently realise within smaller geographical areas or particular clinical specialisations ‘macro’ policy aims.^15^ Nevertheless, there is value in considering the role of policy in ‘meso’ or organisational environments wherein ePrescribing improvement have been achieved apparently in the absence of uniform national ePrescribing capabilities.^15^

### Optimising ePrescribing in hospitals project

**Figure 2.**
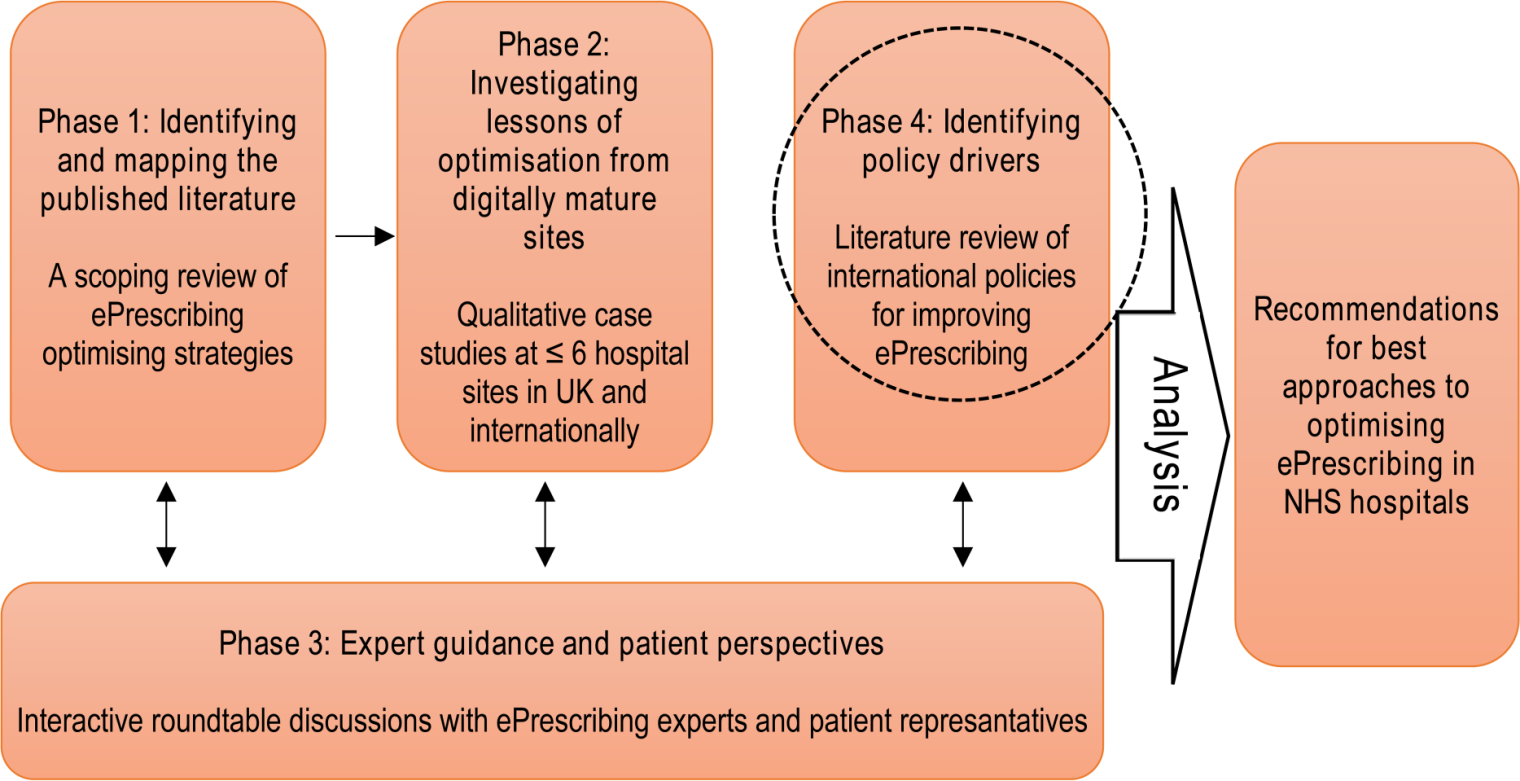
Optimising ePrescribing in hospitals (eP Opt) project overview.^21^

The study discussed here is the last phase in a larger programme of research within the Optimising ePrescribing in Hospitals (eP Opt) Project funded by the National Institute for Health Research (NIHR) (see Figure 2). In this project, we have identified and studied the strategies of those hospital sites wherein there is considerable experience of implementation and subsequent configuration and improvement of ePrescribing systems.^21^ We have done this via a scoping review of scientific literature reporting optimisation of ePrescribing,^10^ in depth case studies of digitally advanced hospital sites in the UK, the US and Europe and expert roundtables with policymakers and systems users.

During the course of the eP Opt project, we realized that most of the literature describing ePrescribing policies focused on implementation, rather than optimisation of ePrescribing systems. Therefore, this review of policy and academic articles relating to policies on optimisation of ePrescribing systems looks to synthesise insights on how policy environments can potentially support improvements to already implemented ePrescribing systems in UK NHS hospitals. We set out to answer the following key questions:

1. Are there examples of policy successfully targeted and implemented to improve ePrescribing in hospitals?
2. What is the context in which these policies have been implemented?
3. Are there applicable lessons for UK policy on optimising hospital ePrescribing?

## Methods

### Overview

We originally set out to search the literature in the manner of a systematic scoping review, which was intended as a sort of reconnaissance of a field of literature.^22^ However, one of the first challenges we encountered was that of finding sources of literature with policy targeted at optimisation of ePrescribing specifically. We were unable to find any database from which we could obtain an international overview of optimisation specific policy. Therefore, we decided to broaden our search and conduct the study in two phases.

### Phase one: Database search

We searched Medline, Embase, NIH and Google Scholar databases using keywords related to ePrescribing and policy (Appendix 1). We focused on recent articles (produced between 2010 and 2020), to capture optimisation. We considered the last review of eHealth carried out for the NHS Connecting for Health's Evaluation Programme as we have done across the project to indicate the end of the first phase of ePrescribing both in the UK and internationally, which would focus mostly on implementation.^10^ Once articles were consolidated, we removed duplicates and applied the following inclusion and exclusion criteria (Table 1) to identify articles that were considered for in-depth analysis. The articles were screened by two reviewers, first by their title and abstract, and then the full text. Disagreements between reviewers were resolved through consensus. We extracted the details of policies on optimising ePrescribing including their purpose, scope, specialisation and focus of the policy intervention on the medication management process. We took an open approach to this so provided that a policy was mentioned it could be at a national, local, organisational, or relating to specific areas of clinical practice or the implementation of particular technologies into the ePrescribing process. As our aim was to capture all the policies associated with optimisation of ePrescribing, we did not carry out a quality assessment on the selected articles.

**Table 1.** Inclusion and exclusion criteria for research papers identified through database search.

Inclusion criteria	Exclusion criteria
Primary articles that describe policies that are associated with the optimisation of an ePrescribing system. The ePrescribing system and healthcare context must be broadly relevant to UK NHS hospitals. The article should be set in a high-income country, as defined by the Organisation for Economic Co-operation and Development (OECD).^19^	Article does not address policy levers associated with the optimisation of an ePrescribing system. Article describes a healthcare context that is not applicable to learning for UK NHS hospitals. The country of the study is not within the OECD. The article was published prior to 2010.

### Phase two: Review of ePrescribing policy documents

As the focus of the articles retrieved from the database search were on the intervention, rather than the policies, and also few in number, we decided to broaden our search to other documents referring directly to policies intended to improve existing ePrescribing capabilities whilst drawing upon literature and reports that provided an overview of policies across different countries. We searched for grey literature, policy documents, reports and official government and institution level communiques in OECD countries and on the National Institute of Health website in the US. As part of the roundtables carried out within the eP Opt project we have consulted with policy, clinical and IT experts (see Figure 2). This along with published literature directed us towards a number of countries wherein there was a history of experience in eHealth and ePrescribing policies.^6,23^ We initially attempted a targeted search of countries for policy documents, but this proved difficult because of language barriers and differences in how the process of optimising ePrescribing in hospitals was described. Therefore, we looked specifically at policy documents related to national level eHealth plans in OECD countries and documents form the NIH website relating to optimising CPOE systems. This led us to look for sources, which could provide a comprehensive international overview of policies on ePrescribing. Figure 1 depicts the focus of policy in which we were interested.

Unfortunately, we did not find a resource that covered all OECD countries, but we were able to find a resource which provided high-level health profiles on the 28 member states of the EU in 2019.^24^ However, whilst the individual OECD Country Health Profiles 2019 provided useful information on various aspects of eHealth, the information on ePrescribing was variable and digital health activities were not covered consistently within all Country Health Profiles. Knowledge of countries such as the UK and the Netherlands, suggested that ePrescribing initiatives at local and national level were not always captured.^25^ In the UK, the extent of work done in individual Trusts, some of which we know to be advanced in ePrescribing practices and processes was, similarly, not covered. Therefore, we do not assume that the OECD Country Health Profiles provided an exhaustive record of optimisation of ePrescribing policy in every country. Nevertheless, the reports were helpful in building up a wider picture of how ePrescribing was being encouraged by policy at a national level.

### Approach to analysis

We classified our findings by the target of the policy intervention in the ePrescribing process and the level at which the policy was enacted. When classifying according to the policy intervention, we considered several models described in literature, including those described by Marceglia et al.^26^ and Bell et al.^27^ We eventually used the model described in Figure 3, as it captures the range of actual policy interventions attempted in hospital ePrescribing and as such was more suited for our article.

The Nuffield Report, ‘Achieving a digital NHS’ identified three levels of policy intervention: the ‘macro’, which aim at changes at the level of the national health system; the ‘meso’, which is organisation based and the ‘micro’, which is on the level of one part of the system or specific technology.^15^ We used this to separate the different policies we encountered. We drew up a typology of policies that govern optimisation of ePrescribing systems in OECD countries, which have broadly comparable legislative standards and income level. For example, in the research literature, which was dominated by the US, the focus of interventions could be described as ‘micro’ or ‘meso’, mainly reflecting policies implemented or enacted at the level of particular technologies or within a given hospital. These papers also referred in some cases to legislation and associated strategies for the national level coordination for ePrescribing programmes or ‘macro’ level policies. In the OECD documents what we most consistently found described were ‘macro’ level or national level policies.

## Results

From Phase one of our study, we identified 11 articles pertaining to policy intervention at macro, meso or micro level (Table 2). In Phase two, we found policy documents from 14 OECD countries.

**Table 2.** Summary of articles retrieved through database search.

Reference	Country	Policy level	Specialisation	Focus of optimisation policy	Key takeaways
Bain et al.^29^	UK	Micro	Diabetes	Lack of flexibility for insulin in ePrescribing systems Improving patient involvement through insulin self-management	17% with ePrecribing systems did not prescribe insulin electronically ePrescribing safety features for insulin were suboptimal Prescribing of variable doses based on carbohydrate intake not allowed
Chaffee et al.^28^	USA	Micro	General	Conflict resolution for CDSS via a Clinical Lead Group	CDSS design decisions to reflect guidelines published by the Institute for Safe Medication Practices
Chaturvedi et al.^32^	USA	Meso	Intravenous clinical integration (IVCI)	Integrating ‘smart’ infusion pumps in Electronic Health Records (EHR) Hospital wide standardisation of drug libraries and workflows	Automation can ‘increase small-scale precision while leading to larger-scale errors’ May be necessary to limit the precision of documentation to high-risk medications
Cortelyou-Ward et al.^35^	USA	Meso	General	Medication reconciliation at transition of care	Federal legislation and guidance have increased use of CPOE in conjunction with CDSS in reconciliation process for complex medication needs
Finnerty et al.^36^	USA	Micro	Psychiatry	Restrictions to polypharmacy in psychiatry	Nonauthoritative policies enforced via monitoring rather than hard stops by the ePrescription system
NCPDP^31^	USA	Macro	Drugs with narrow therapeutic index	Algorithms calculating cumulative daily doses from product exposure Review safety related policies for order entry (e.g. review medications before entering new orders)	CPOE evaluation should use standardised tools (e.g. LeapFrog) Alerts should be targeted to avoid ‘alert fatigue’
Rodriguez et al.^30^	USA	Micro	General	Implementation of formulary restrictions	Subsequent policies needed to address full range of drug interchange scenarios (e.g. dose restrictions, and non-formulary drugs)
Wakefield et al.^37^	USA	Micro	General	Automated Drug Cabinets (ADC) and Bar-coded Medication Administration systems (BCMA)	Safety and quality improved via changes to ADC operating mode and integration of BCMA
Walroth et al.^38^	USA	Micro	General	Reduce clinically insignificant smart-pump alerts by implementation of a standardized, consensus driven process for smart-pump drug library	Patients’ involvement in policy making at a state level for safety drive around smart pump technology use
Wilson et al.^33^	USA and UK	Macro	General	Comparing legislation around digital health including ePrescribing for the USA and UK	Proper governance of interoperability needed to ensure patient safety aims of EHR implementation are achieved
Wright et al.^34^	USA	Micro	General	Change of CDSS as a cloud-based service from local implementation Interoperability through Continuity of Care Document standard	Emphasised that clinical decision making can only be supported rather than dictated by CDSS

### Phase one: Database search

The PRISMA diagram appears in Appendix 2. One paper was a comparison of policy between the UK and the US,^33^ while most of the articles (*n* = 9) were from the US.^28,30–32,34–38^ The other article was from the UK.^29^ Overall, therefore, we are able to provide information on policies related to optimisation only in the UK, mainland Europe and the US.

Figure 3 shows the ePrescribing process alongside examples of policy interventions targeting optimisation from the literature.

### Levels at which policy is enacted

Here we describe the levels of policy at the finer grain at which we encountered them in the policy and research literature review with impact on the particular stages of the ePrescribing process (Figure 3). Policy interventions, especially at a national level, do not always map directly onto particular parts of the process as shown in Figure 3. We have grouped the interventions into macro, meso and micro policy levels as mentioned above. Micro level interventions would be likely to target a very specific aspect of the ePrescribing process, meso level several parts and macro level could be aimed at the whole process by targeting infrastructure and interoperability for example (see Table 3).

**Table 3.** Typology of policy level of interventions for optimising ePrescribing systems.

Level	Aim(s)	Potential barriers to successful application of optimisation policy	Examples	Potential to impact/aspect of the ePrescribing process impacted
International/cross border Macro	Sharing health information Interoperability Accessibility	Lack of interoperability standards	epSOS: Successful pilot project shared electronic Prescriptions within EU countries HL7 standards	
National Macro	Interoperability Creating resource for health care and research	Lack of infrastructure	Norway: Dignio – widespread clinical adoption of ePrescribing tools	
Regional Meso	To produce a standardised drug library for smart pump use across six care providers in Indianapolis – and reduce alerts	Lack of care provider coordination	Succeeded in reducing the number of ‘insignificant’ alerts’	Formulary Prescribing/ordering Order communication
Hospital Meso	To increase adherence to drug-specific formulary restrictions	Rapid installation by vendor – lack of tailored optimal functionality	Computerised drug order entries (DOEs) – need for continuous oversight	Formulary Prescribing/ordering
Specialisation Micro	Reduction of Insulin prescribing errors	Lack of functionality in ePrescribing systems	Leapfrog Objective tools to evaluate ePrescribing systems	Prescribing/ordering
Process Micro	Monitoring access to controlled medications	Increased workload for staff and increased costs	Barcode medication administration and Automated Dispensing Cabinets used in conjunction to improve patient safety, accountability, and monitoring	Prescribing/ordering Order communication

### Policy targets at the micro and meso levels

We found evidence in the literature of micro level policies aimed at solving specific problems or addressing particular processes.^28–30,34,36–38^ These targeted policies included digitising the prescribing of particular drugs categories, the integration of different aspects of digital equipment and on specific processes such as medication reconciliation. There were policies aimed at addressing concerns about the capability of ePrescribing systems in handling certain situations. For example, drugs with a narrow therapeutic index like paracetamol, gentamycin and digoxin require precise cumulative dose calculations across all routes of administration and all preparations, which are beyond the capability of most ePrescribing systems.^31^ Taking paracetamol as a model, the National Council for Prescription Drug Programs issued national guidelines with several recommendations to optimise ePrescribing systems in prescribing such medications.^31^ In one US example patients had input into policy making at a state level for a specific safety drive around smart pump technology use.^40^ Here the issue being addressed was primarily to control alerts, which would create noise in the system and give rise to adverse drug events.

Bain et al.^29^ found that even among hospitals that use commercial ePrescribing systems 17% used paper prescriptions for insulin. Some of the issues, which lead to the use of paper prescriptions were mitigated by adopting a policy of prescribing insulin only by brand name and directing physicians to organisation specific protocols in prescribing.^29^ Similarly, dispensing controlled medications to patients require special vigilance, authorisation and maintenance of chain of custody, which needs to be replicated in the ePrescribing workflow. Some hospitals in the US had a policy of using Automated Dispensing Cabinets (ADC) in ‘profile mode’ so only drugs that had been pre-approved by a pharmacist could be dispensed to a patient.^37^

#### Connected equipment

A comprehensive ePrescribing system interfaces with other types of medical equipment in order to optimally deliver its services. CDSS may interface with laboratory devices for dose recommendations, or with smart pumps to directly deliver precise drug doses, or with ADCs for validating the patient with his order. Such interfacing requires interoperability standards, as well as standardisation of workflows, drug libraries and care protocols.^32^ The problem of alerts related to smart pump use was targeted so as to ensure the benefits of the intended role of smart pumps in infusion-based medication errors were not undermined.^38^ These policies aimed for standardisation and interoperability between connected equipment. Improved configuration of alerts supplied as standard by the vendor, were also a target for optimisation within the hospital setting.^32,38^

#### Transformation of care/medication reconciliation

Many ePrescribing systems employ formulary restrictions to optimise patient safety. Medications may also be limited due to availability and cost reasons. However, such restrictions may lead to some patients not being able to continue their long-term medication whilst admitted to the hospital. Some hospitals have policies to reconcile these existing medications with the hospital formulary in a transparent manner with pre-set action plans.^30^ Another study dealing with reconciliation described how a patient led group worked to enact various levels of policy including legislation to ensure interoperability of secondary and primary care systems and integration of the CPOE and CDSS with the EHR. The aim was to enable ‘patient-centred’ recommendations based on clinical guidelines and accurate data on the patient's medical history.^35^

### Phase two: Review of ePrescribing policy documents

#### Macro level focused policies

There were only a few examples of macro level policy explored in the research literature. However, we wished to explore all levels of policy that could target the optimisation of ePrescribing in hospitals. For this reason, we have included insights also from the OECD Country Health Profiles for 2019 for the, then 28, EU countries.^24^ These are reports on various aspects of the health system of a country, including some information on eHealth. We noted that a national level ePrescribing system was explicitly referred to in 12 cases, as part of a larger national level eHealth programme. Fragmentation due to the existence of different health providers and insurers, within many European countries was evident, echoing the situation for US health systems.^41^ Among other challenges for optimisation posed by this is the barrier it creates for sharing patient data across health care settings.^6^ However, in some cases, including Belgium, Czech Republic, Italy and Norway, these issues were tackled by dedicated eHealth policies or government bodies. Norway has an eHealth directorate, which is part of the Ministry of Health. Germany has pilot ePrescribing projects but does not yet have a sustained national programme for universal roll-out and adoption in all hospital sites.^42^

In Iceland despite widespread use of EHRs, integration across the seven health regions and between public and private sectors clinics remained a challenge.^43^ Development of eHealth had been slow in the Netherlands and there was no standardised electronic patient record, which would enable national level eHealth to scale up.^27^ Despite this, there has been significant work on infrastructure in the Netherlands, where use of EHR and ePrescribing by clinicians has been reported to be amongst the highest in Europe.^44^ Across Europe, there was explicit mention of eHealth strategies or initiatives as part of wider health care strategies at national level in 19 of the 28 EU member states and none at all of ePrescribing.^24^ In Czech Republic, Finland and Germany, fragmentation in planning and implementation of eHealth and health policy generally were noted, resulting in some cases in a low level of ePrescribing uptake being reported amongst clinicians.^24,41,42^ There was evidence that within some countries there was a growing digital maturity gap between different hospitals, whereas in others such as Denmark and Norway there was more even development of capacity nationally.^41^

### Coordinated policy initiatives for improvements to ePrescribing

For Denmark and Norway, there was evidence of high levels of consistency in terms of the roll out of digital health services and related improvements across the entire country. In particular, Norway appeared to have high levels of coordination in terms of national policies to support improvements to eHealth generally.^24^ The Norwegian Directorate of eHealth (NDE) was established in 2016 to develop national eHealth policies and coordinate both geographically and across primary and secondary care, and other healthcare bodies. One of the policy initiatives rolled out at a national level involved allowing patients access to their hospital ePrescribing records.^24^

### National initiatives for patient access and involvement

Relevant policy for patients took broadly two forms. The first was patient representation in bodies tasked with drawing up or consulting on policy. Eight of the national Country Health Profiles for the (then) 28, member states (i.e. Belgium, Czech Republic, Denmark, Iceland, Malta, Norway, Poland and Spain) mentioned patient involvement in health policy or eHealth initiatives explicitly. In Spain the Strategic Framework for Primary and Community Care was created in April 2019 by the Ministry of Health, autonomous communities, professional organisations and patient organisations.^24^ In Denmark, organisations (represented by the Association of Danish Patients) had been involved in drawing up recommendations by the Medicine Council around access to medicines, which was established in early 2017.^45^

The second type of policy encouraged patient access to their eHealth, including, ePrescribing information. In Denmark, the national EHR system allowed access to individual patient medical records for patients and health professionals for primary care and secondary care.^24^ As part of the Norwegian eHeath initiatives, patients could access a wide range of personal health information, including their ePrescriptions.^24^ However, at a national level there were few countries that offered patients access to their health data in Europe (Denmark, Estonia, Finland, France, Iceland, Norway, Scotland, Sweden and, recently, England). Recently the U.S. Department of Health and Human Services finalised two rules that allowed patients to access their data through any third-party application of their choice using a secure, standards-based application programming interface (API).^46^ At the time of writing this has yet to be rolled out.

### Difficulties of mapping policy directly onto the ePrescribing process

In one study in Indianapolis, a not-for-profit patient coalition sought to operationalise the National Patient Safety Goals, the purpose of which is to improve patient safety by focusing on specific safety problems. These goals are set yearly by the Joint Commission, a US-based non-profit patient safety organisation, which operates at a national level in consultation with experts and stakeholders.^38,44^ The Joint Commission evaluates a range of health care programmes, such as ambulatory care and offers certification for standards in, for example, integrated care. It has been involved in an initiative to address alert fatigue for alarmed medical devices.^47^ It worked with Indianapolis Coalition for Patient Safety Smart Pump Alert Fatigue Workgroup across six different health providers operating in the state. The impact of national level legislation and policy in respect of standards and access to national level resources such as drug code information has been seen as key to outcomes such as adherence.^47^ Moreover, national level infrastructure and policy has been found to contribute to the ability of patients to view their medication information.^44^ National legislation mentioned only in the US literature as relevant to the optimisation work being carried out included the HITECH Act (2009) and Patient Protection and Affordable Care Act (2010).^33^ It is not straight forward therefore, to map policy directly onto aspects of the ePrescribing process as some policy seeks for a wider improvement in eHealth and ePrescribing infrastructure, which indirectly will impact the potential to improve the process.

### Insights relevant to the UK context

One stated aim was to consider how this compares to the UK, in order to distil relevant lessons. The typology of policy intervention shows the different strategies used to implement policy in order to make improvements to ePrescribing systems. The UK has an NHS that is similar in function across the four devolved nations, with near universal coverage of the population, split into hospital trusts in England, health and social care trusts in Northern Ireland and regional health boards in Scotland and Wales. Potentially, therefore, policies at national, regional, specialisation, hospital and process level can be relevant in the UK context. Despite the NHS being a national level organisation different trust will have a degree of choice in how systems are implemented and optimised and varying budgets with which to achieve their aims.^48^

Allowing patients access is an improvement to the system in that it allows the accuracy of information held in patient records and dealing with prescriptions to be checked by patients themselves. In both Denmark and Norway this was coordinated at a national level, which appeared to allow innovations to the ePrescribing system, such as patient access to their ePrescribing data.

## Discussion

### Principal findings

We found evidence of a number of policy initiatives operating at macro, meso and micro levels, but these were typically not strategically aligned with little in the way of empirical evaluation to allow sharing of lessons within or across countries. In a number of the research articles from our initial literature search we encountered policy being used to target particular problems, or aspects of the ePrescribing process, for example, medicines reconciliation or the use of smart pumps.^30,38^ We were interested in learning how policy interventions aimed at optimising ePrescribing both rested upon and encouraged existing digitally maturity in the hospital setting. We considered how eHealth policy has been employed to address problems or push forward improvements to ePrescribing at a national level. We looked at barriers to both digital maturity in eHealth systems and in relation to attempts to improve ePrescribing systems. There is evidence of uneven roll out of ePrescribing in many national settings, which hampered some attempts at optimisation.

### Strengths and limitations

In a number of US sites, there was a dynamic interplay between national and local policy actors not all of whom were directly within national or state government. In several European countries similarly the national infrastructure does not exist for a nationwide rollout of eHealth strategies, meaning that implementation of ePrescribing provision is not consistent and this is reflected in how policy is adapted.^41^ In a number of cases the technological capacity may exist within a country, but a national rollout of ePrescribing improvement is thwarted by the piecemeal nature of health providers and insurers and a lack of coordination across geographical health regions.^42,43^ A number of countries are characterised by pockets of ePrescribing activity, pilot projects and local initiatives. Access of patients to their health data is in place in a small number of countries, but there is little evidence available to suggest that this is the case for ePrescribing information beyond Denmark and Norway.

### Interpretation in the context of the wider literature

Due to the obstacles described above, we were unable to access a source that would give us an overview of policy documents relating specifically to the optimising of ePrescribing. We make no claims to our search being systematic as access documents were based on their availability. Individual OECD Country Health Profiles 2019 provided useful information on the presence of eHealth and ePrescribing.^24^ However, ePrescribing was not the focus of these documents and information on ePrescribing was variable and digital health activities were not covered consistently or exhaustively. The lack of quality assessment was another limitation of this analysis.

Our study was limited to countries of the OECD due to constraints of time, budget and the original aims of the wider eP Opt project. However, we have laid a foundation for further research that expands to global policy on ePrescribing.

### Impact of the COVID-19 pandemic on optimising ePrecribing

During the writing and submission of this paper, health systems have faced an unprecedented challenge in the shape of the COVID-19 pandemic. Whilst many have discussed the possibilities for managing COVID using eHealth technologies,^49,50^ few have directly explored policy interventions aimed at making improvements to the ePrescribing system. Early findings exploring the related area of governance point to how a relaxation in governance processes enabled, in some cases, hospitals to utilise ePrescribing capacity in a responsive way.^51^ This relaxation saw greater use of telehealth and upscaling of patient portals, for example. However, removal of the ordinary checks and balances may need to be revisited when designing policy for ePrescribing in the medium to longer term.

### Implications for policy, practice and research

Some countries have opted for a decentralised approach to the governance of ePrescribing implementations, whilst others have opted for a centralised approach. In some cases, a variety of local actors are applying and adapting national or regional policy initiatives related to but not necessarily specifically addressing ePrescribing. The Wachter Review stated that it would be a mistake to move too far away from the centralised approach seen in the NHS National Programme for IT (NPfIT), even though the perceived failure of the overall programme was often attributed to this approach.^52^ Simultaneously, international efforts around standardisation for ePrescribing in relation to nomenclature, for example, SNOMED-CT continue to be important.^15^ Overall, the picture though does appear to remain one of leading institutions in an otherwise irregular landscape in terms of eHealth infrastructure. Much activity is still focused upon rollout and implementation of eHealth infrastructure and this is reflected in policy. Our findings are consistent with other research that notes enormous variability in digital maturity of health systems generally and ePrescribing in particular, as well as difficulties in balancing the national against the local.^13^ It is interesting, moreover, to consider the potential tension between a benchmarking approach, which allows leaders to emerge, and the attempts at standardisation and uniform policy, which appears to signal towards a universal rollout of similar systems.

## Conclusions

Calls which were made more than a decade ago, for better standardisation at both national and international level^6^ appear to be some distance from what has been achieved in relation to eHealth more generally. The recent Deloitte report on digital transformations in health suggested that interoperability be a priority, as well as a robust ‘health IT infrastructure’ and governance framework.^41^ It has been suggested that the best way to achieve this is at the national or even international level.^6^ This latter fits in with the notion of interoperability across national systems, which remains an important goal for the NHS.^53^

From this initial scoping of literature on policy related to the optimisation of ePrescribing it would appear that a coordinated approach may pay dividends in terms of increased confidence to attempt strategies such as patient access to their own records. Such initiatives undoubtedly require not only a robust technical infrastructure but a corresponding policy drawing upon experience of using the systems and a corresponding confident approach to governance. We found examples, of conditions, which created barriers to certain types of optimisations, despite existing policy, across all policy and research literature, for example, if sufficient infrastructure did not exist to allow rollout of ePrescribing and related eHealth initiatives. We also found examples of optimisations, which flourished given a combination of a supporting infrastructure and policy initiatives. These included micro level or problem focused policies, which were designed to solve particular issues occurring within a local site or a particular type of drug.

## Appendixes

### Appendix 1

[Table table4-20552076221085074]

**Table table4-20552076221085074:**

Search terms
1. exp Medication Systems/ or Drug Information services/ or adverse drug reaction reporting systems/ or clinical pharmacy information systems/ or Technology, Pharmaceutical/ or Pharmaceutical Services, Online/ or Clinical Pharmacy Information Systems/ or drug therapy, computer-assisted/ or Medical Order Entry Systems/ or Electronic Prescribing/ or Decision support systems, clinical/ or Decision support techniques/ or Decision making, computer assisted/
2. (E-prescri* or Eprescri* or Electronic prescri* or "Electronic Transmission ADJ2 Prescription*" or "Computer* Physician Order Entry" or CPOE or EMAR or "electronic medication administration record" or "electronic medicines administration record" or "Hospital electronic prescribing ADJ2 medication administration" or "Hospital electronic prescribing ADJ2 medicines administration" or HEPMA or "Medic* Order Entry Systems" or ADR or "adverse drug reaction* report* system*" or "Medication system*" or "Medicine* system*" or "Medicine* administration*" or "Medication* administration*" or "Clinical decision* support" or CDSS or "decision support technique*" or "medic* management solution*").tw.
3. (Drug Prescriptions/ or Medication therapy management/) and (cell phone/ or Smartphone/ or Mobile applications/)
4. ((ePrescribing or e-prescribing or prescribing) adj3 (app* or mobile* or Smartphone*)).tw.
5. (eprescribing or e-prescribing or prescri*).tw. and (Software/ or software.tw.)
6. (Robot* and (dispens* or pharmac*)).tw.
7. ("Integrated electronic prescrib*" or "Automat* dispens*" or "Robotic prescri* dispensar*" or "Closed loop prescri*").tw.
8. 1 or 2 or 3 or 4 or 5 or 6 or 7
9. (Optimi?ation or Optima* or "System optimi?ation").tw.
10. "Quality ADJ2 healthcare"/ or Quality improvement/ or Safety/ or Patient safety/ or Efficiency/ or Clinical audit/ or Data reuse/ or cost benefit analysis/
11. (Quality or Improv* or Efficiency or audit or "Data reuse" or "System iteration" or Workaround* or "Continuous cycle* ADJ2 improvement" or "Reporting system*" or "Data quality monitoring" or "Critical incidence reports" or "End-user feedback" or Upgrad* or "Benefits reali?ation" or "Investment analysis").tw.
12. 9 or 10 or 11
13. 8 and 12
14. (policy/ or policies.tw.)
15. 13 and 14
